# Women’s participation in university management in Spain: The case of Andalusian universities

**DOI:** 10.1371/journal.pone.0307170

**Published:** 2024-08-09

**Authors:** María Josefa Rodríguez-Baiget, Carmen Corpas-Reina, Alexander Maz-Machado, Gema del Rosario Linde-Valenzuela

**Affiliations:** Universidad de Córdoba, Córdoba, España; Instituto Tecnologico Autonomo de Mexico, MEXICO

## Abstract

This study addresses the presence of women in the management of Andalusian public universities, Spain. The aim of this study is to determine the representation of women in the administration and management of the administrative units of Andalusian public universities at faculty and department level, as well as to identify the distribution of university administration in terms of gender of managers in university centres according to the different macro-areas of the division of scientific knowledge. The method used was a descriptive study with quantitative and ex post facto values. A sample was selected from all public universities in the Autonomous Community of Andalusia, which represent 20% of all public universities in Spain. Information was collected from all academic units and the gender of each responsible administrator was determined. The data were deposited in a virtual repository. The results revealed that, in general, there is a disproportion in the predominance of male managers and administrators compared to the number of women involved in university management tasks in Andalusia. Imbalances in gender representation at different levels of management were observed, reflecting the inequalities reported in the literature. This study confirmed the existence of gender biases in university management, aligning with existing literature, which highlights the importance of addressing gender inequalities from a holistic perspective. The findings underline the importance of continuing to work on promoting gender equality in university management through multi-factorial approaches and concrete actions.

## Introduction

In Spain there are 76 universities in operation, 50 of them are public, and 20 are private. During the academic year 2021/2022 in Spain 13011 people took part of the teaching and research staff collective (PDI). The 54% of PDI are civil servants, and 43,35 of them are women [1]. According to Hernández & Pérez [2] in 2018 more than 50% of women were focus on the occupational groups of associate professors and doctoral assistants, administrative levels that entail less responsibility. They are the lowest step of the professional pyramid in the university sector.

The autonomous community of Andalusia, in Spain, has a population of 8.5 million people. In order to provide university education for this population, Andalusia has 10 public universities in which 201637 students study [3]. At the same time, these higher education centres are organised into faculties, higher technical schools, etc., and in all these entities there are staff who carry out administrative functions, which are essential for the optimum functioning of the training programmes of each university degree.

While in recent years women have begun to have a greater presence in universities (as students, professors, researchers, and managers), several studies point out that the role of women is disadvantageous in university management, and therefore the gender distribution in academic work continues to widen [4–6]. As O’Keefe & Courtois [7] point out in the international scientific community, there is concern about the access of women to professor or senior management positions. Many promotion criteria and mechanisms have been shown to reflect gender norms, favouring hegemonic male behaviours [8, 9].

In the scientific literature, the term "glass ceiling" was coined in order to signal all of those guidelines and rules unidentified by the organizations that make it difficult for women to have access to the high positions of management.

According to Andrew [10], the gender of inequality is internalized in our society, and the fact of carry out awareness campaigns in order to turn things around is not enough. The administration and government agencies must be taken specific actions and concrete measures.

The gender gap in science refers to the inequality between women and men in terms of representation, opportunities, and recognition in the scientific field. This inequality is manifested in several aspects, such as the lack of representation of women in leadership positions, the wage gap between women and men for the same work, and the lower visibility and recognition of the contributions produced by women scientists: Lower representation, difficulties in accessing funding and resources, pay inequality, and lower visibility and recognition. In terms of the degree of representation, in many scientific disciplines, women are under-represented in leadership and decision-making positions [11, 12] There is also a significant gap in the representation of women in fields such as engineering and computer science [13–15].

In terms of difficulties in accessing funding and resources, the literature and sources consulted indicate that women scientists often face obstacles in accessing funding opportunities and support for research compared to their male counterparts [16, 17]. This is compounded by pay inequality which implies that also in scientific settings, women receive lower salaries than their male counterparts for work of equal value and expertise [18, 19]. Finally, women scientists enjoy lower visibility and recognition Women’s contributions in science are often not recognised in the same way as men’s, which can affect their career advancement and influence in the scientific community [20–22].

The international literature has studied the challenges and boundaries faced by women to access to the top positions in business and in the university. According to Gallego-Morón and Matus-López [23], there are 3 major aspects that directly affect in the glass ceiling:

- Personal boundaries: it refers to the difficulty of find the balance between personal live, family and professional life.
- Organizational boundaries and structural issues: it refers to the existence of a misogynist context that materializes in sexist and discriminatory attitudes towards women.
- Social boundaries: it characterized by the coexistence in a historical context of discrimination against women, where there is a differentiated socialization based on gender, among other things.

Achieving equality and empowering all women entails approving and strengthening policies and laws applicable to their promotion, so as to ensure the full and effective participation of all women with real opportunities to exercise their leadership at all decision-making levels in political, economic and public life; this is reflected in the goals of the 2030 Agenda for the development of the Sustainable Development Goals (SDG) [24].

The SDG 2020 report reflects international commitments to promote gender equality and some progress in areas such as high representation of women in politics but warns that the COVID-19 pandemic has undermined this progress. As of January 1, 2020, as achievements, the report highlights that the representation of women in parliaments in the various countries has improved slightly reaching 24.9%, up from 22.3% in 2015. Information from 133 countries indicates that women currently have men better access to decision-making positions at the local level, occupying 36.3% of elected positions in local deliberative bodies. Only 13% and 15% of countries, respectively, have achieved gender balance (40% or more) in legislative bodies in national parliaments and local government. This increase seems to be due to the measures taken by each government in legislative terms to establish certain gender quotas in various sectors of society.

The European Union, for its part, makes explicit the need for inclusive and diverse leadership to deal with the decision-making of an increasingly complex society, thus strengthening democracy and effective policy-making by redistributing power and influence. The report that the European Union presented in 2022 on the state of equality between women and men, in the framework of the new Strategy for Gender Equality 2020–2025 [25], presents a balance of the situation of the countries of the European Union regarding gender equality, confirming that women are still very under-represented in decision-making positions in all areas, since there are still few women in managerial positions in companies, high courts or public bodies; even though at the lowest levels there is parity and they are highly qualified, they still do not assume management and leadership positions in organisations [26].

Only a minority of women reach the highest positions, reflecting the inequalities between Member States. Among the causes of female under-representation shown in this report are traditional gender roles and stereotypes, the unequal sharing of domestic and care responsibilities, as well as political and labour cultures that favour long working hour’s incompatible with the care responsibilities traditionally assigned to women. These factors discourage and limit participation of women in politics and public life and, consequently, hinder gender equality in decision-making, leading to a flagrant pay gap, what Jabbaz, et al. [27] call *grietas de discrecionalidad* (in English, discretionary cracks) in spaces and micro-discriminatory practices within the university structure.

According to the report *Científicas en Cifras* [28], by the Unidad de Mujeres y Ciencia (UMYC) (in English, Women and Science Unit) of the Government of Spain, there has been some improvement in the presence of women in management positions. In 2020, in the unipersonal bodies of the universities there were 23% of women in rector positions and 50% of women managing an Oficina de Proyectos Internacionales (OPI) (in English, International Project Office), at the level of vice-rectors there is almost a situation of gender balance (42% in 2020).

Gender gaps persist, such as the fact that women do not participate fully and equally in decisions that affect the scientific system (23% of female rectors and managers of research institutes), which is why it is still necessary to commit and involve all the agents of the science, technology, and innovation system to continue with specific lines of action that allow for the eradication of inequality.

In this sense, the European Union report [25] has introduced important changes in the field of research and innovation, with initiatives aimed at strengthening gender equality in training and education, dissemination of gender-sensitive research and innovation, among which we highlight the requirements from April 2021 to obtain funding from the different programmes (Horizonte Europa, Europa Creative MEDIA, Erasmus+, etc.) that must include a gender equality plan in the application with the consequent impact on the selection of the projects that are evaluated and the awarding of funds.

In one of the first studies on management and gender in Spain [29], that the deterioration of dedication to research activities is one of the main consequences of performing university management tasks. of the main consequences of performing university management tasks. Likewise, it was found that women that during their professional careers, women postpone the possibility of accessing management positions, due to the dedication and management positions, due to the dedication and attention to the family as well as to the prioritization of the professional development of the partner. professional development of the partner.

The challenge towards a true culture of gender equality in universities remains and it is essential to address it from a comprehensive co-educational model [30] that permeates the entire university structure. Preliminary studies with reference to the presence of women in Andalusian universities with respect to the level of research groups has been pointed out imbalances and negative bias towards women [31, 32].

The objective of this study is to determine the representation of women in university faculties and centers. The aim is to identify the distribution of university management and administration in terms of the gender of the managers in the university centers according to the different macro-areas of scientific knowledge division.

## Material and methods

This is a descriptive study with quantitative and ex-post facto values. A sample of all public universities in the Autonomous Community of Andalusia was selected. These universities represent 20% of all public universities in Spain.

The universities analysed are University of Almeria (UAL), University of Cadiz (UCA), University of Cordoba (UCO), University of Granada (UGR), University of Huelva (UHU), International University of Andalusia (UNIA), University of Jaen (UJA), University of Malaga (UMA), University of Pablo de Olavide (UPO) and University of Seville (US). These universities have a total of 131 centres (Faculties, Higher Technical Schools, etc.). Some universities have campuses in different cities, such as the UGR, which has campuses and faculties in North Africa, in Ceuta and Melilla; the UCO also has a campus in the city of Belmez, to name a few. The management private centers that are attached to universities were also considered.

The instrument used for data collection in the study consists of a grid that includes the following categorical and quantitative variables. Categorical variables: Name of university, position and gender of the governing team, name of each faculty, name of each head of polytechnic school and department, gender of each member of the dean’s team, gender of each dean and head of polytechnic school, gender of each head of department. Quantitative variables: number of faculties or polytechnics, number of vice-deans, number of vice-rectors, number of women in each of the above groups.

The data obtained have been deposited in the virtual repository Helvia of the University of Cordoba (https://helvia.uco.es/xmlui/handle/10396/27534).

For the data collection, in September 2022, all information related to the management and direction of the faculties, technical colleges and departments that are accessible from the websites of each university and each faculty and department were accessed.

Subsequently, the gender of each of the administrators responsible for each faculty or college of engineering was determined.

**Fig 1:**
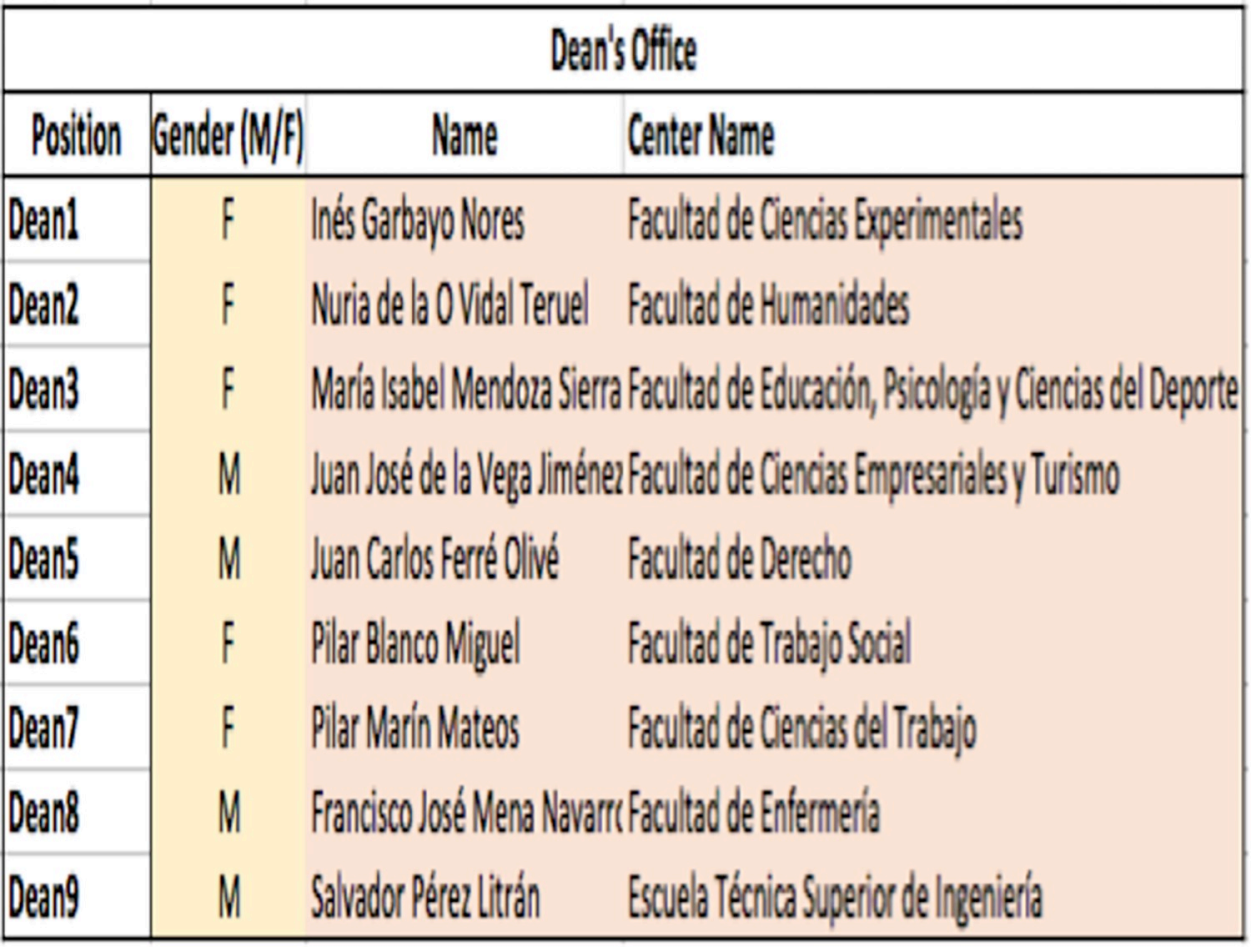
Example of a data collection template for each university.

Once the information had been identified on the website of each university and faculty, it was manually dumped onto an Excel spreadsheet (Fig 1). The data was manually cleaned and then the biological gender of each of the university and faculty managers was determined according to the first name indicated on the website. This methodology has already been implemented in similar studies [33].

## Results and discussion

When all 131 administrative management centers of the Andalusian universities were analyzed for each university, the result is that the universities of Granada and Seville are the institutions with the highest number of Faculties or Higher Technical Schools with 26 and 25 respectively. Both institutions have 38.93% of all these administrative institutions in the Andalusian university system. Among all the women who manage these centres, the UCO has the highest percentage of women in the management of educational centres belonging to Andalusian universities; in this university the 60% of these centres are managed by women. This is followed by the UPO, where women management represent 57,14% of the administration (Table 1). If we analyze the difference between men and women, 62.6% of the total positions of Dean or Director are held by men, while women hold 37.4%. In three of the universities, there is most women over men in these positions; these are UCO (60%), UPO (57,14%) and UHU (55.56%). In the other eight universities, men are in the majority. The great imbalance between men and women in management at the US, UIA and US universities is remarkable. In the US, men manage 84% of the centers, in the IAU 75% and 70.59% in the AMU.

**Table 1:** Management of engineering schools or faculties by universities and gender in Andalusia.

UNIVERSITY	PDI	Gender			
		Female	%	Male	%
**Univ Almeria**	957	4	40.00	6	60.00
**Univ Cadiz**	1760	7	43.75	9	56.25
**Univ Córdoba**	1533	6	60.00	4	40.00
**Univ Granada**	3709	10	38.46	16	61.54
**Univ Huelva**	934	5	55.56	4	44.44
**Univ Int. Andalucía**	*	1	25.00	3	75.00
**Univ Jaen**	987	3	42.86	4	57.14
**Univ Malaga**	2707	5	29.41	12	70.59
**Univ Pablo Olavide**	1038	4	57.14	3	42.86
**Univ Sevilla**	4400	4	16.00	21	84.00
**Total**	18065	49	37.40	82	62.60

* The university does not have any research teaching staff dependent on the university.

With the data in Table 1, the Pearson correlation can be made because the variables PDI and % are normal (p = 0.139 and p = 0.200 respectively) with the Kolmogorov-Smirnov test. Subsequently, the Pearson correlation coefficient was calculated, for which the value of -0.578 was found.

**Fig 2:**
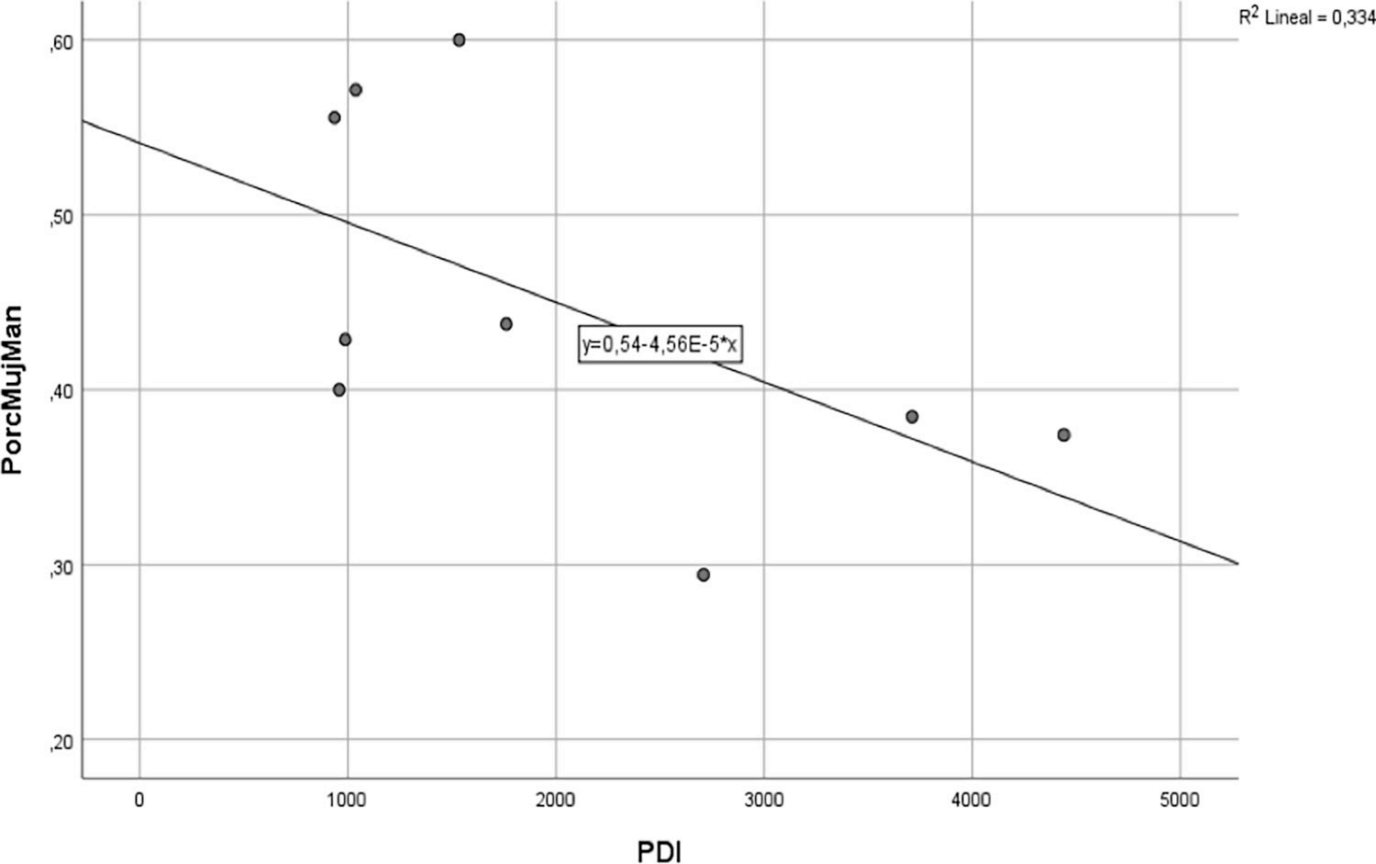
Scatter plot assessing the relationship between the number of PDI and the percentage of women.

The resulting value is -0.578, which means that there is an inverse linear correlation of moderate intensity between the two variables. This means that, as the number of teaching and research staff in a university increases (size of the university), the representation of women in management positions decreases (Fig 2). This suggests that larger universities tend to have lower proportions of women in these roles.

**Fig 3:**
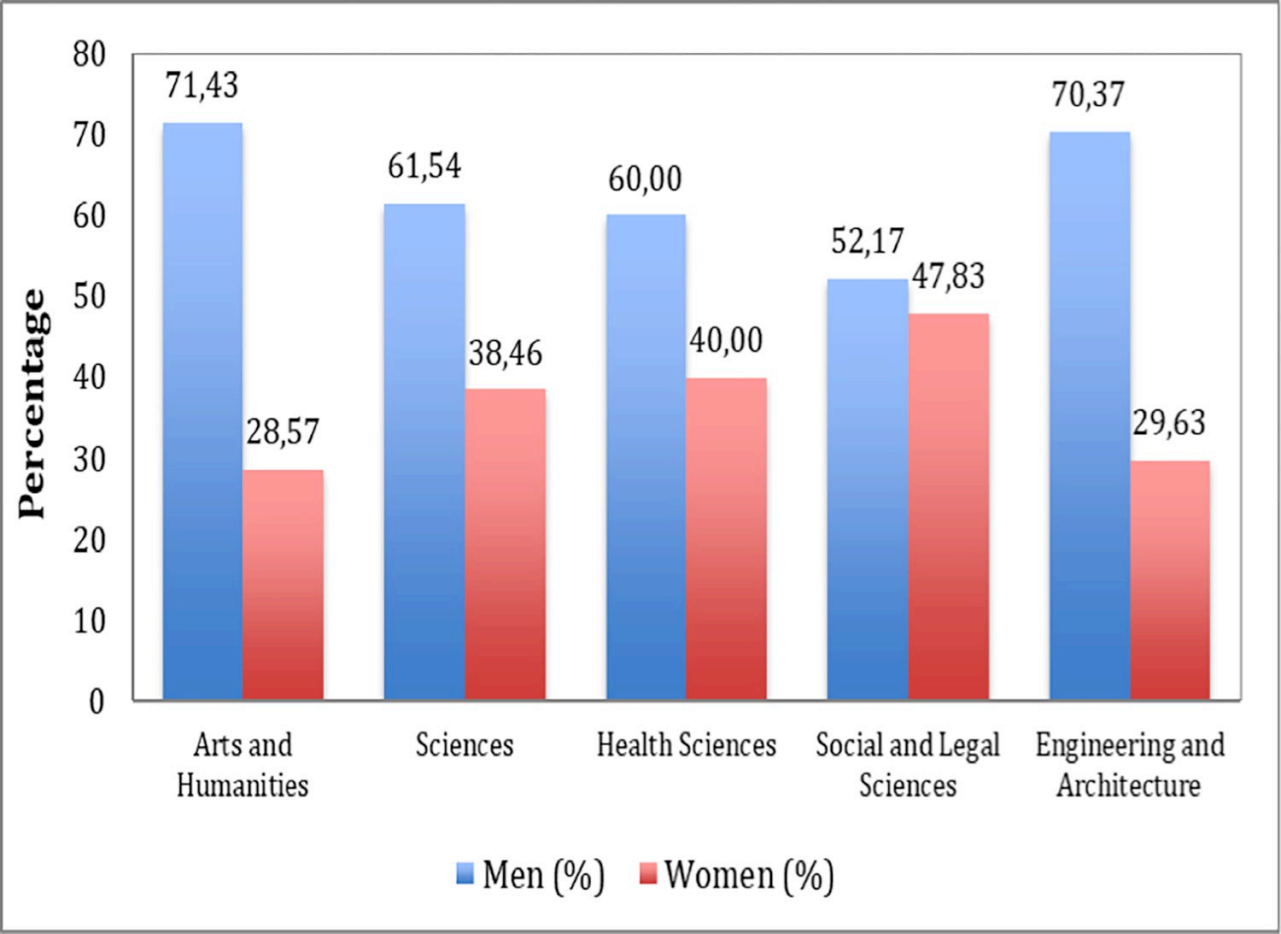
Percentage of men and women responsible for the management and administration of Faculties or Higher Technical Schools.

When observing the behaviour of management according to gender in the different macro-areas, it is evident that in the university faculties linked to the Health Sciences, male representation is greater than that of women in the highest management positions, with men leading 60% of the faculties compared to only 40% led by women (Fig 3).

Looking only at the management of technical colleges, 66.67% of them are headed by men compared to 33.33% headed by women. When analysing what happens in the faculties of education, a reversal in the gender percentages is obtained, because in these, women are deans in 71.43% of them compared to men who manage 28.57%.

When analysing the management of other types of centres (doctoral schools, directors of generic centres), 85.71% are the responsibility of men compared to only 14.29% who have a woman as director.

With respect to the staff in charge of the secretariat of faculties or colleges; in the Andalusian universities is employed principally by men (Table 2).

**Table 2:** Responsible for the secretariat of engineering schools or faculties by university and gender.

UNIV	Gender				
	Female	%	Male	%	Total
**Univ Pablo Olavide**	3	42.8	4	57.1	7
**Univ Córdoba**	6	60.0	4	40.0	10
**Univ Granada**	12	46.1	14	53.9	26
**Univ Almería**	5	50.0	5	50.0	10
**Univ Cádiz**	3	18.7	13	81.2	16
**Univ Huelva**	6	66.6	3	33.3	9
**Univ Int. Andalucía**	2	50.0	2	50.0	4
**Univ Jaén**	4	57.1	3	42.8	7
**Univ Málaga**	6	35.2	11	64.7	17
**Univ Sevilla**	10	40.0	15	60.0	25
**Total**	57	43.5	74	56.5	131

When comparing the gender of the deans or directors with respect to those in charge of the secretariat of these centers, it is observed that only in the UCO is there absolute equality. However, in the UIA and US universities, it is observed that women increase their presence in percentages of 25% and 24%, respectively, in relation to the position of dean.

Another aspect that has been analyzed is the number of vice deans in each university and the representation by gender in these management positions in the second level.

Globally, there is a balance in terms of gender, only in the universities of Almeria and Cadiz are there significant differences. In the first one, two thirds of the vice-deans are women, while in the second one, 60.21% are men (Table 3).

**Table 3:** % of vice-deans by university and gender.

UNIV	No. of vice-deans	Gender			
		Female	%	Male	%
**Univ Pablo Olavide**	21	12	57.1	9	42.8
**Univ Córdoba**	23	11	47.8	12	52.1
**Univ Granada**	91	42	46.1	49	53.8
**Univ Almeria**	20	15	75.0	5	25.0
**Univ Cadiz**	31	12	38.7	19	61.2
**Univ Huelva**	34	17	50.0	17	50.0
**Univ Jaen**	33	19	57.5	14	42.4
**Univ Malaga**	63	37	58.7	26	41.2
**Univ Sevilla**	87	39	44.8	48	55.1
**Total**	403	204	50.6	199	49.4

All the universities in Andalusia have 92 vice-rectorates that administer and manage the different policies and initiatives of each university. Of these vice-rectorates, 54.3% are headed by men and 45.65% by women. There are no vice-deans at the University International Andalusia. The reason for this is that according to their statutes, it is only dedicated to postgraduate training and therefore does not require this administrative segmentation.

The University of Seville has the highest number of women heading the vice-rectorates, 77.8% compared to 22.22% headed by men. At the opposite extreme is the University of Granada, where women head the lowest number of vice-rectorates, only 25% compared to the 75% that are headed by men (Table 4).

**Table 4:** % of vice-rector by university and gender.

UNIV	Gender				Total
	Female	%	Male	%	
**Univ Pablo Olavide**	4	44.4	5	55.5	9
**Univ Córdoba**	5	50.0	5	50.0	10
**Univ Granada**	2	25.0	6	75.0	8
**Univ Almeria**	3	33.3	6	66.6	9
**Univ Cadiz**	5	50.0	5	50.0	10
**Univ Huelva**	6	66.6	3	33.3	9
**Univ Int. Andalucía**	2	33.3	4	66.6	6
**Univ Jaen**	4	40.0	6	60.0	10
**Univ Malaga**	4	33.3	8	66.6	12
**Univ Sevilla**	7	77.7	2	22.2	9
**Total**	42	45.6	50	54.3	92

On the other hand, the University of Cordoba and the University of Cadiz show a balance between the number of male and female vice-rectors.

The findings underscore the gender disparities prevalent in administrative management positions across Andalusian universities, with some institutions and sectors showing more progress towards gender balance than others.

## Conclusions

The study revealed a disproportion in the predominance of male managers and administrators compared to the number of women involved in university management tasks. Although progress has been made in many of the universities in Andalusia to equalise the presence of women in academic and administrative management positions, inequalities continue to exist.

According to Luque-Martínez et al [34] there is an existence of vertical discrimination, because the higher up the professional hierarchy, the fewer women are present.

In the management of Faculties or Higher Technical Schools, there are clearly some cases where a bias in favour of men is evident (e.g., at the University of Malaga and the University of Seville).

In the analysis by macro-areas in these administrative units, the gender bias towards women is observed in Arts and Humanities, and Engineering and Architecture. The Social and Legal Sciences macro-area is the one where there is the greatest balance between men and women in terms of the management of the centres.

In general, in the vice-rectors, the gender patterns that occur at the level of the Faculties or Higher Technical Schools are reproduced. However, in some universities, progress has been made in achieving equality or reducing the gender gap as much as possible (University of Cordoba, University of Cadiz, and University of Pablo de Olavide).

This study has shown that, despite the increase in state laws and regulations that have been approved to fight against gender discrimination, there is still a long way to go before these measures are applied in the reality of universities, at least in the case of Andalusian universities.

The results indicate that further research should be carried out to identify the factors that lead to this discrimination in management and administrative roles at the deanery level in Andalusian universities as a whole. It is also evident that in certain universities there are large imbalances in terms of vice-dean positions.

These results are somewhat difficult to understand since all the universities studied have equality units which are supposed to ensure the promotion of gender equality policies at all levels and in all areas of the university.

We can conclude that much remained to be done in order to reduce the gap between men and women with respect to university management in higher education institutions in Andalusia.

In relation to the gender of those who occupy the position of vice rector, there is evidence of an improvement with respect to what another study found for universities in Spain a decade ago, when women represented only 34.4%, compared to the results of the present study, where they represent 45%.

The next step in this line of research is to extend the analysis to other administrative units such as the departments and auxiliary units present in Andalusian universities (Equality Unit, university defender, etc.).

Confirmation of Gender Biases: the presence of gender biases in university management was confirmed, which is aligned with the literature consulted that mentions the existence of challenges and barriers for women in leadership and management roles in academic settings [22, 35, 36].

We also identified imbalances in gender representation at different levels of management, reflecting the inequalities reported in the literature. For example, the study shows differences in the representation of women at the level of deans, directors, vice-deans and vice-rectors, in line with imbalances reported in previous research [12, 20, 35, 37].

The study has reflected the importance of addressing gender inequalities from a holistic perspective, including personal, organisational and societal aspects. These findings are in line with the literature, which highlights the complexity of factors influencing gender representation in leadership positions at university level [38, 39].

These results support the predicted results found in the scientific literature by emphasising the need to promote concrete policies and measures to empower women and promote gender equality in decision-making. The study echoes the literature by proposing the need for specific actions to address the gender gap in university management [11, 21].

The results of this study reinforce the importance of continuing to work on the promotion of gender equality in university management through multifactorial approaches and concrete actions [19, 20, 40, 41]. These insights could inform policy and initiatives aimed at promoting gender equity within academic leadership.

We also consider that it is necessary to extend the study to other Spanish universities to determine whether these results are similar to those of Andalusia or whether these patterns are present in other universities in Spain.
